# When Medication Isn’t Enough: Personality Pathology and Adaptive Outcomes During Pharmacological Treatment of Adult ADHD

**DOI:** 10.1192/j.eurpsy.2026.11467

**Published:** 2026-07-31

**Authors:** P. Jacobsson

**Affiliations:** Sahlgrenska Academy, University of Gothenburg, Gothenburg, Sweden

## Abstract

**Introduction:**

Adult attention-deficit/hyperactivity disorder (ADHD) is highly heterogeneous, encompassing variable cognitive, emotional, and interpersonal difficulties. While stimulant medication effectively reduces core symptoms, many adults continue to experience substantial difficulties in everyday functioning. Personality pathology—defined by disturbances in self- and interpersonal functioning (Criterion A) and maladaptive trait expression (Criterion B)—may help explain these differential outcomes.

**Objectives:**

To examine how dimensional models of personality pathology influence symptom change, treatment adherence, and adaptive outcomes during pharmacological treatment of adult ADHD, and to consider implications for resource allocation based on severity.

**Methods:**

This research program integrated meta-analytic, cross-sectional, and longitudinal data from naturalistic clinical samples (n = 246–330) receiving stimulant therapy. Participants completed the LPFS-BF 2.0, PID-5, ADHD symptom measures (ASRS/CSS), and the WHODAS 2.0. Linear mixed-effects and survival models tested the effects of personality dysfunction and maladaptive traits, controlling for stimulant dose and treatment duration.

**Results:**

Higher general personality dysfunction predicted greater and more persistent impairment (β ≈ .45, p < .001) despite symptomatic improvement. Maladaptive domains—particularly Negative Affectivity, Detachment, and Psychoticism—were most strongly associated with limited adaptive gains. Disinhibition, especially its facets Distractibility, Impulsivity, and Irresponsibility, was uniquely related to residual ADHD symptoms, while interpersonal traits such as Intimacy Avoidance and Deceitfulness predicted premature discontinuation of medication.

*Note: A figure illustrating standardized β values and 95 % confidence intervals for personality domains predicting functional impairment is available upon request.*

**Conclusions:**

Findings highlight the heterogeneity of treatment response in adult ADHD and indicate that personality pathology constrains the extent to which pharmacological treatment enhances everyday adaptation. Incorporating dimensional personality assessment into ADHD care may support stratified allocation of resources and integrated treatment approaches that combine medication with targeted psychological strategies.

**Disclosure of Interest:**

None Declared

